# Mechanisms that Trigger a Good Health-Care Response to Intimate Partner Violence in Spain. Combining Realist Evaluation and Qualitative Comparative Analysis Approaches

**DOI:** 10.1371/journal.pone.0135167

**Published:** 2015-08-13

**Authors:** Isabel Goicolea, Carmen Vives-Cases, Anna-Karin Hurtig, Bruno Marchal, Erica Briones-Vozmediano, Laura Otero-García, Marta García-Quinto, Miguel San Sebastian

**Affiliations:** 1 Unit of Epidemiology and Global Health, Department of Clinical Medicine and Public Health, Umeå University, Umeå, Sweden; 2 Grupo de Investigación de Salud Pública, Universidad de Alicante, Alicante, Spain; 3 Departamento de Enfermería Comunitaria, Medicina Preventiva y Salud Pública e Historia de la Ciencia. Universidad de Alicante, Alicante, Spain; 4 CIBER de Epidemiología y Salud Pública (CIBERESP), Barcelona, Spain; 5 Institute of Tropical Medicine, Antwerp, Belgium; 6 Faculty of Medicine, Universidad Autónoma de Madrid, Madrid, Spain; National Institute of Health, ITALY

## Abstract

**Background:**

Health care professionals, especially those working in primary health-care services, can play a key role in preventing and responding to intimate partner violence. However, there are huge variations in the way health care professionals and primary health care teams respond to intimate partner violence. In this study we tested a previously developed programme theory on 15 primary health care center teams located in four different Spanish regions: Murcia, C Valenciana, Castilla-León and Cantabria. The aim was to identify the key combinations of contextual factors and mechanisms that trigger a good primary health care center team response to intimate partner violence.

**Methods:**

A multiple case-study design was used. Qualitative and quantitative information was collected from each of the 15 centers (cases). In order to handle the large amount of information without losing familiarity with each case, qualitative comparative analysis was undertaken. Conditions (context and mechanisms) and outcomes, were identified and assessed for each of the 15 cases, and solution formulae were calculated using qualitative comparative analysis software.

**Results:**

The emerging programme theory highlighted the importance of the combination of each team’s self-efficacy, perceived preparation and women-centredness in generating a good team response to intimate partner violence. The use of the protocol and accumulated experience in primary health care were the most relevant contextual/intervention conditions to trigger a good response. However in order to achieve this, they must be combined with other conditions, such as an enabling team climate, having a champion social worker and having staff with training in intimate partner violence.

**Conclusions:**

Interventions to improve primary health care teams’ response to intimate partner violence should focus on strengthening team’s self-efficacy, perceived preparation and the implementation of a woman-centred approach. The use of the protocol combined with a large working experience in primary health care, and other factors such as training, a good team climate, and having a champion social worker on the team, also played a key role. Measures to sustain such interventions and promote these contextual factors should be encouraged.

## Introduction

Men’s intimate partner violence (IPV) against women is a global public health problem and has devastating effects on the health and wellbeing of women and children [1–3]. In the EU-27, IPV affects between 20% and 25% of adult women who have ever had an intimate partner [4]. Aside from potential death, IPV is related to physical and psychological injury, negative health behaviours, chronic conditions, reproductive health problems, and mental health problems [3, 5, 6].

The health system, especially primary health-care services, can play a key role in preventing and responding to IPV. First-line health-care facilities are the public institutions most frequently accessed by women exposed to IPV [2, 7, 8]. However, encounters between women exposed to IPV and health-care providers are often not satisfactory [9, 10], and a number of barriers preventing health-care providers from responding to IPV have been documented [11–13].

According to the current World Health Organization’s guidelines, a good health care response to IPV includes all or an important part of the following actions: being aware to recognise the possible signs and symptoms, providing health-care assistance and registration, informing affected women about available resources, coordinating with other sectors, and raising public awareness. These actions should be carried out within a woman-centred approach, namely, ensuring privacy and confidentiality, and being non-judgemental and supportive of the diverse needs that each woman might have [2, 14].

Women exposed to IPV do access primary health care centres (PHCCs) in Spain. PHCCs are made up of multidisciplinary teams of family doctors, nurses, social workers, midwives and paediatricians. Every person older than 14 is assigned to one PHCC where the same family doctor and nurse will follow him/her for several years, enabling a trusting relationship to be built. A cross-sectional survey conducted with 11,000 adult women in Spain found that women exposed to IPV have a two times higher likelihood of visiting PHCCs than those who have never been abused [8]. However, it is unclear how many of those cases are actually detected, and of those detected, what type of response they receive.

In Spain, the “Gender Based Violence Law” enacted in 2004 specifically addressed the responsibilities of the health sector [15]. Grounded in this Law, the 17 decentralised regional Spanish health systems have developed interventions aimed at: 1) developing protocols to guide health providers’ response to IPV, 2) training health-care professionals, and 3) developing and implementing an IPV monitoring system [16, 17]. However, our previous studies with primary health care professionals in this country have pointed out huge variations in the way individual professionals responded to IPV. Individual level factors, such as the professionals’ motivations, backgrounds, ideologies, and professionalism, played a key role in the way that professionals responded to IPV. Team level factors-managerial style, team working style, team learning structures- were also important in shaping how individual professionals responded to IPV. In fact, it was observed that differences in responses to IPV are not only found at the individual professional level, but also at the team level: there are primary health care teams that respond better than others [16].

Building on the knowledge gained through these previous studies and following a realist evaluation design (see the section below for a further description of realist evaluation), we developed a programme theory- the theory behind an intervention, namely why, how and which components of an intervention trigger certain mechanisms within individuals (or groups of individuals) to achieve certain outcomes. The initial programme theory pointed out that:

An intervention to improve the health-care response to IPV that consists of: 1) developing protocols and guidelines based on state-of-the-art knowledge, 2) the training of health professionals—on a voluntary basis—aimed at raising the awareness of providers, transferring know-how, and convincing them to respond to IPV, and 3) weak monitoring of the implementation of policy, legitimises and supports intrinsically motivated health-care professionals, but does not promote internalised motivation among those who are not intrinsically motivated. However, other institutional interventions are able to promote internalised motivation to deliver health care with a woman-centred approach. When such an approach is internalised, integrating an IPV response follows naturally among those who are internally motivated. For those who are less motivated, the team-learning, team-working and referral structures promoted by such an approach gives increased self-security and might promote increased internalisation in the long run.

Personal attributes, including a sensibility to issues of IPV, often based on personal activism (feminism), facilitate uptake of the intervention, as they enhance intrinsic motivation and may facilitate the internalisation of the values underlying the policy. Professional attributes were also key, social workers being shown to have an increased readiness over the rest of the professionals.

Team attributes that enable the uptake of such policies include a person-centred approach and a strong primary health-care approach, while conducive organisational attributes include a management style that values team learning, team work and individual initiative. Safe spaces for reflection and case discussion and the presence of experienced providers facilitate team learning and contribute to increased self-confidence in less-experienced providers. Therapeutic groups for women and adequate referral networks also provide further support for professionals dealing with IPV. The adoption of a broad perspective, for instance, by adopting ‘women’s malaise’ as the entry point, allows more health professionals to detect and refer cases of IPV.

This study tests the previously described programme theory in 15 PHCC teams located in four different Spanish regions- Murcia, C Valenciana, Castilla-León and Cantabria with the aim of identifying the key combinations of contextual factors and mechanisms that trigger a good primary health care center team response to IPV.

## Methods

The study followed a multiple case study design [18]. The cases included 15 primary health care teams located in four different Spanish regions. The four regions- Murcia, C Valenciana, Castilla-León and Cantabria- were different in terms of size, socio-demographic indicators and also the implementation of IPV interventions within the health care system [16]. Information was collected from January 2013 until March 2014. We combined realist evaluation and qualitative comparative analysis to assess the connections between contextual and intervention factors, mechanisms generated at the team level, and outcomes- in terms of a good PHCC team response to IPV.

### Combining realist evaluation and qualitative comparative analysis to identify cross-case patterns

Realist evaluation is a type of theory-driven evaluation that aims to ascertain why, how and under what circumstances programmes succeed or fail [19–22]. Realist evaluation begins with the formulation of the theory behind the development of a programme, known as the programme theory. The programme theory is formulated on the basis of previous research and/or knowledge and the experience of the stakeholders involved in the intervention, and states how the intervention is supposed to trigger change. Context-Mechanisms-Outcome configurations (CMOs) that describe patterns or causal chains are the basis of the programme theory. The programme theory is then tested through empirical research of cases in which the programme has been implemented. The analysis of data collected in these cases serves to refine the preliminary programme theory [19, 23–25]. Quantitative and qualitative data are usually collected and combined to identify these CMO configurations or patterns. Qualitative comparative analysis is both an approach and an analytical technique to study cases as configurations of conditions. It allows the combination of both a sufficiently in-depth exploration of individual cases and the identification of patterns that are connected with different outcomes. Qualitative comparative analysis uses Boolean algebra to assess the extent to which a configuration of conditions explain outcomes [26–34].

In this study, we used qualitative comparative analysis mainly as an analytical technique to explore the conditions (Context, Intervention and Mechanisms) that were sufficient and/or necessary to make PHCC teams respond adequately to IPV (the outcome measured). The potential context-mechanism-outcome configurations to be tested in selected cases emerged from the programme theory presented previously.

In line with realist evaluation principles, the potential context-mechanisms-outcome configurations contained in the programme theory were tested in 15 purposively selected cases- primary health care teams. The inclusion criteria for PHCCs was to be a public PHCCs, and in addition we aimed to include different profiles of PHCCs in terms of perceived responsiveness to IPV and location. Six of the teams were selected because they were perceived as responding better to IPV, while nine were perceived as responding on average. All of the 15 teams were integrated by family physicians, nurses, midwife(s), and paediatrician (s). All of the teams but one had a social worker. The size of the teams ranged from 13 health professionals to 38. Eight of the teams were located in the provincial capital or a large city, while seven were located in smaller cities, towns or villages. Further descriptions of the 15 PHCC teams can be found in S1 Table.

### Data collection

Qualitative and quantitative data were collected from each case by four of the authors (IG, EBV, LOG and MGQ). In an iterative process, the data to be collected was guided by the programme theory, while at the same time as the data collection process, the data collection tools and data to be collected were refined through gaining familiarity with the cases. Throughout this process, a set of variables to assess the different conditions (context, mechanisms) and outcome contained in the programme theory were identified. Table 1 describes the elements of the programme theory that were assessed, the variables to assess them, and the tools used.

**Table 1:** Description of conditions and outcome. Elements of programme theory (PT), descriptor and data collection techniques and tools.

Elements of PT	CMO in focus	Descriptor	Data collection	Abbreviation used
Training on IPV received	Intervention-Context	Mean number of hours of training received	Questionnaire PREMIS	Train
Knowledge/use of IPV protocols	Intervention-Context	% of professionals who have read the protocol	Questionnaire PREMIS	Prot
PHC approach	Context	Mean number of years working in PHC	Questionnaire PREMIS	PHC
Team’s organization and climate	Context	Observer´s perception on team´s climate and organization based on: regularity and participation in meetings, recreational activities, existence and perception of team coordinator and environment.	Qualitative information: observation and interviews.	Clim
Social worker’s (SW)role	Context	Observer´s perception regarding the role of the SW: existence or not, number of days per week working, professionals´ perceptions, SW´s interest on IPV.	Qualitative information: observation and interviews.	SW
Self-efficacy	Mechanism	Mean score on self-efficacy: capability to asks new patients, being comfortable discussing IPV, ability to detect IPV	Questionnaire PREMIS	SE
Woman centredness	Mechanism	Mean score on opinions on victim’s understanding.	Questionnaire PREMIS	VU
Knowledge	Mechanism	Mean score on knowledge	Questionnaire PREMIS	Know
Perceived preparation	Mechanism	Mean score on perceived preparation	Questionnaire PREMIS	PPrep
Time-climate/workings on IPV	Mechanism	Mean score on opinions on workplace issues- intersectorial response, time management, privacy-, and staff constrains- opinions on health professionals responsibility with IPV	Questionnaire PREMIS	TeamIPV
Response to IPV	Outcome	Mean score on Practice issues- number of new diagnosis, clinical inquiry, detection of signs and symptoms, actions taken, referrals	Questionnaire PREMIS	Pract

Qualitative data were collected through interviews and observation. Interviews were conducted with health care professionals and professionals working on specific services directed towards women exposed to IPV in the vicinity of the case. For healthcare professionals the inclusion criteria were: to be working in the PHCC as a family doctor, nurse, social worker, paediatrician, midwife, or other relevant health care professional. In order to capture diversity of experiences both health care professionals interested on IPV and those less interested were interviewed. For professionals working on IPV institutions the inclusion criteria was: to be working on an institution specialized on IPV located in the vicinity of the PHCC explored. In PHCCs where it was safe and feasible to interview women who had been exposed to IPV, the criteria for selecting these women was: women who had been exposed to IPV and who have attended the PHCC being explored.

Quantitative information was collected through the Physicians’ Readiness to Manage Intimate Partner Violence questionnaire (PREMIS), which has been adapted and validated in Spain [35, 36]. Data collected through the application of the PREMIS questionnaire helped to gather information on several conditions (Table 1). For data collected in the PREMIS questionnaire see S2 Table.

The number of participants and data collected in each case are summarised in Table 2.

**Table 2:** Data collected. Summary of data collected in each case.

Region	Health center and regional level	Interviews with health professionals within the team	Other interviews (IPV^1^ services and women)	PREMIS^2^ questionnaires returned
Cantabria	General	16	1 (IPV services)	27
	Vegas	9	3 (IPV services)	19
	Salinas	9	1 (IPV services)	14
	Indias	10	2 (IPV services)	12
	Regional		2	
Castilla Leon	Mares	10		10
	Angeles	9		12
	Avecilla	9	1 (woman) 1 (IPV services)	10
	Regional		2	
Murcia	La Virgen	17	4 (women) 3 (IPV services)	25
	El Campo	14	2 (women) 1 (IPV services)	18
	Mora	9	3 (IPV services)	19
	Cristina	10	3 (IPV services)	21
	Regional		4	
C Valenciana	Santos	9	1 (IPV services)	11
	Rios	11	4 (IPV services)	20
	Castillo	13	1 (woman) 2 (IPV services)	23
	Naranjo	10	1 (IPV services)	24
	Regional		1	

^1^ IPV services refers to services specialized in supporting women exposed to intimate partner violence in terms of legal, social, economic and/or psychological issues

^2^ PREMIS refers to the Physicians Readiness to Manage Intimate Partner Violence Questionnaire. It is a comprehensive and reliable tool to measure physicians’ preparedness to manage IPV [35, 36].

The researcher(s) spent at least one week on each case- more time was spent on the first four cases, when the data collection tools were fine tuned.

### Data analysis

Responses to the PREMIS questionnaires were entered into a data base and analysed using Epi Info. Total scores were calculated for nine aspects: 1) perceived preparation, 2) perceived knowledge, 3) actual knowledge, 4) practice issues, 5) opinions on work-place issues, 6) opinions on constraints, 7) opinions on self-efficacy, 8) opinions on victims’ understanding, and 9) opinions on victim autonomy. Total scores for each dimension were calculated for each professionals and for each case (PHCC team). For calculating the scores we followed the formulas provided by the developers of the instrument [36]. Thick descriptions were produced to gain familiarity with each case [18]. The qualitative information collected provided information on some of the conditions that were not covered in the quantitative data. Using the qualitative and quantitative information contained in the thick descriptions of the cases, numeric values were given to each of the conditions/outcomes assessed, and a table was produced.

The table emerging from the analysis of the thick descriptions of the cases had to be calibrated in order to conduct a qualitative comparative analysis using fuzzy sets [37]. Fuzzy sets are used when the causal conditions and outcomes are multichotomies, namely, they vary by level of degree [29]. We did the calibration with the help of the software programme fzQCA, assigning the highest, middle and lowest values. A raw table with calibrated values can be found in Table 3.

**Table 3:** Raw table. Calibrated raw table with assessed conditions and outcome for each case.

	Train	Prot	PHC	Clim	SW	SE	VU	Know	PPrep	Team IPV	Pract
**La Virgen**	0.29	0.71	0.72	0.77	0.95	0.73	0.46	0.76	0.89	0.95	0.6
**El Campo**	0.15	0.31	0.48	0.95	0.95	0.83	0.76	0.12	0.53	0.83	0.81
**Mora**	0.29	0.14	0.54	0.32	0.47	0.05	0.23	0.39	0.14	0.05	0.02
**Cristina**	0.26	0.06	0.29	0.05	0.47	0.2	0.05	0.05	0.65	0.23	0.11
**Santos**	0.3	0.13	0.05	0.77	0.47	0.15	0.45	0.26	0.49	0.26	0.05
**Naranjo**	0.15	0.15	0.12	0.05	0.47	0.26	0.38	0.15	0.3	0.3	0.05
**Rios**	0.62	0.79	0.32	0.05	0.05	0.44	0.31	0.32	0.95	0.47	0.47
**Castillo**	0.86	0.72	0.08	0.32	0.95	0.83	0.7	0.3	0.84	0.71	0.63
**Salinas**	0.95	0.05	0.86	0.32	0.17	0.92	0.92	0.95	0.93	0.67	0.89
**Vegas**	0.46	0.84	0.67	0.77	0.81	0.78	0.87	0.88	0.68	0.77	0.76
**Indias**	0.49	0.09	0.3	0.95	0.17	0.2	0.37	0.46	0.47	0.48	0.09
**General**	0.49	0.43	0.17	0.77	0.47	0.42	0.95	0.82	0.29	0.58	0.49
**Angeles**	0.05	0.95	0.87	0.05	0.17	0.55	0.72	0.68	0.8	0.46	0.75
**Mares**	0.59	0.86	0.95	0.77	0.47	0.95	0.58	0.22	0.91	0.24	0.95
**Avecilla**	0.22	0.49	0.71	0.32	0.17	0.31	0.82	0.46	0.05	0.36	0.22

The raw table was imported into the software fzQCA to assess the combinations of contextual factors and mechanisms that lead to the outcome of IPV response. The following conditions were assessed in terms of potential mechanisms: Self-efficacy, Victim understanding- woman-centredness, Knowledge, Perceived preparation, and Team workings on IPV. In terms of intervention and contextual factors, the following conditions were assessed: Protocol use, Training received, Experience with PHC, Team climate/organisation, and Champion social worker. In terms of outcomes, the team response to IPV cases measured through the mean score on Practice issues was assessed. A description of each condition and outcome can be found in Table 1.

The fzQCA software was used to evaluate the different combinations of conditions that were connected with positive outcomes, namely, better responses to IPV. This process aligns with the retroduction approach used in realist evaluation, whereby the observed outcomes are explained by looking into the mechanisms and context elements, namely, what are the factors that are present among the “best cases” and are absent among the “poorer cases”.

In order to test the different combinations of conditions, a truth table was produced. The truth table displays all of the possible combinations of causes leading to the outcome, which in this study, was a good response to IPV. From the emerging truth table, the inconsistencies were eliminated- configurations of conditions with less than one case were excluded, and the outcome was reset to 1 if consistency was higher than 0.8. A standard analysis was applied, and the intermediate solution formula was chosen- based on logical reduction, but retaining conditions that theoretically contribute to an explanation [38, 39]. The solution formula depicts those combinations that are more relevant to produce the outcome. Usually, more than one combination of conditions emerges, and for each of them, consistency and coverage scores are given. Consistency represents the extent to which a combination of conditions leads to an outcome and ranges from 0 to 1. If a combination of conditions has a consistency of 1, this means that such combination always leads to the outcome. Coverage represents how many cases with the outcome are represented by a particular combination of conditions. If a combination of conditions has a coverage of 1, this means that this combination is able to explain all of the occurrences of the outcome.

Ethical approval for this study was granted by the Ethical Committee of the University of Alicante (Spain). The study was presented to the regional public health authorities and to the health teams of the 15 PHCCs, who approved its implementation. Written informed consent was sought from all of the participants in the study. Confidentiality was assured and pseudonyms were used for all of the respondents and for the PHCCs.

## Results and Discussion

The truth table is presented in S3 Table, while the solution formula calculated, and its consistency and coverage, are presented in Table 4.

**Table 4:** Intermediate solution formula. Combination of context and mechanisms that lead to good responses to IPV.

Context-intervention		Context-team factors			Mechanisms					Raw coverage	Consistency
			Clim	SW	SE	VU		Pprep	TeamIPV	0.45	0.94
	Prot	PHC			SE	VU	Know	Pprep		0.43	0.97
Train		PHC			SE	VU	Know	Pprep	TeamIPV	0.39	0.93
Train	Prot			SW	SE	VU		PPrep	TeamIPV	0.37	0.90
Train	Prot	PHC	Clim		SE	VU		PPrep		0.32	0.99
	Prot	PHC	Clim	SW	SE		Know	PPrep	TeamIPV	0.34	0.94

Solution coverage: 0.747460

Solution consistency: 0.908289.

The solution formula had a consistency of 0.91, meaning that it produced the outcome most of the time. The coverage was 0.75, meaning that a moderate number of cases with the outcome were represented by this combination of conditions. In summary, 1) these combinations of conditions triggered good team responses to IPV most of the time, 2) there is not only one key combination of conditions leading to good practices, but many different pathways, and 3) there might be other conditions that are important for triggering a good team response to IPV which have not been explored in this study.

The solution formula highlighted the relevance of three mechanisms: self-efficacy, perceived preparation and woman-centredness. The mechanisms of self-efficacy and perceived preparation were present in all of the combinations, meaning that they are necessary for triggering good team responses to IPV. The mechanism of woman-centredness was present in five of the six possible combinations, also denoting its relevance. The mechanism of teams working on IPV was present in four combinations, while knowledge was present in three combinations. None of the mechanisms alone was sufficient to generate the outcome, meaning that they should be combined with other mechanisms and contextual factors in order to generate a good team response to IPV.

In terms of contextual/intervention conditions, the use of the protocol and experience in primary health care were both present in four combinations each, while training, a good team climate and a champion social worker were present in three combinations each.

### Key mechanisms: self-efficacy, perceived preparation and woman-centredness

Perceived self-efficacy concerns “judgements of how well one can execute courses of action required to deal with prospective situations” [40]. The mechanisms of self-efficacy also have been related to higher performance and lower emotional arousal in relation to actual clinical responses to women who have been abused [40, 41].

The qualitative information also supports the relevance of self-efficacy and perceived preparation in triggering a good team response to IPV. Self-efficacy and perceived preparation grow progressively and influence responses, not only at the individual, but also at the team level:

*I think that in the team we have grown*, *every day we are growing*. *Nowadays*, *I do not respond to cases of violence in the same way I did four years ago […] Nowadays*, *I feel more secure [when responding to cases of IPV] as part of a group of people who are really motivated with this topic*. *But even the rest of the team who are not that motivated*, *I can feel that they have grown… […] they are aware that we as a team are responding to IPV; they know that if they detect a case*, *in this team*, *we can respond very well… (Family Physician 1*, *La Virgen*, *Murcia)*

This finding also aligns with Bandura’s notion in regards to how collective efficacy is rooted in self-efficacy and how it influences what people choose to do as a group and the effort they put into it [40].

Self-efficacy and perceived preparation contributed to triggering a good response, while when professionals felt ill prepared, responses were poorer. Among the cases with the lowest scores on practice issues, the participants repeated many times how the professionals felt “unprepared, lacking the skills” (Midwife 1, Avecilla, Castilla-León), “ill prepared to respond” (Nurse 1, Indias, Cantabria), or “with no clarity of our role” (Family Physician 1, Indias, Cantabria). The following quotation from a medical doctor in Indias reflected how professionals on her team felt ill-prepared to respond in a professional way to IPV, although they might be sympathetic with the women exposed to IPV:

*For any other health problem*, *we know how to respond*, *but with IPV*, *we don’t know how to respond in a professional way*. *We respond as anybody would*, *a woman tells us this [that she is exposed to IPV] and we want to help her*, *as anybody would do…*, *but we do not know how to respond in a professional way; I think that we are not handling these cases adequately (Family Physician 2*, *Indias*, *Cantabria)*

Woman-centredness also emerged as a relevant condition for a good response to IPV. The literature states that woman-centredness is a requirement for a good quality health care response to IPV [2]. Although there is not much empirical research backing the effectiveness of implementing a woman-centred-response to IPV, the practitioners working with IPV support such an approach. In addition, the adoption of a woman-centred approach to health has been proven useful in the provision of other health care services, such as reproductive and maternal health care [42, 43].

In the individual interviews, such an approach to IPV was linked to gendered approaches to health care, such as the malaise approach to health that considers somatic symptoms with no identifiable organic cause to be related to contextual, subjective and gender-related factors, and that a purely biomedical approach to health cannot adequately address such symptoms [44, 45]. As the coordinator of one primary health care area in Murcia stated, the earlier implementation of such an approach in some PHCCs in the region facilitated the later integration of a IPV response:

*The women’s malaise approach allowed health professionals to understand that there are other causes of illnesses*, *other social determinants of health*, *such as gender issues…This approach allowed health professionals to feel more confident when managing uncertainty*, *and that facilitated the work when it came to integrating a response to IPV (PHC Area Coordinator*, *Murcia)*

In a more gender neutral way, such an approach was also connected by participants with an overall primary health care approach/family medicine approach that focuses on the individual person-patient and not on his/her concrete health problem:

*I follow the same patients for many days*, *years*, *I know them…*, *that’s the advantage of being a family doctor*: *We know the patient and his/her context […] So*, *a patient might start telling me about symptoms that are not biologically rooted*, *and I know her situation*, *her context*, *so in a couple of minutes*, *I will know what to look for… [Family Physician 1*, *El Campo*, *Murcia)*

### Contextual factors

In terms of contextual and intervention factors, what seems key is to ensure a combination of different conditions—both in terms of intervention components and team level factors- instead of a single “magic bullet” condition. The fzQCA showed that it was mostly the use of the protocol and a good team climate that contributed to better responses. Our findings align with the recommendations contained in the WHO guidelines advocating for multicomponent programmes integrated within existing health-care services [2, 14].

The use of the protocol appeared in four of the combinations of conditions. The importance of the protocol also emerged from the interviews with professionals and observations. A written protocol provided a guide for “essential” actions to be carried out,narrowed down what to do, contributed to reduce uncertainty and to increase self-confidence and self-efficacy, as the PHCC coordinator from Naranjo pointed out:

*[The protocol] I think it works when you are a bit lost and you don’t know what to do*, *when you do not know how to detect [IPV]*. *For us medical doctors*, *it makes us feel more secure*, *when we don’t know about something*, *to follow a protocol where everything is systematised (PHCC coordinator*, *Naranjo*, *C Valenciana)*

The protocol also acted as a reminder, particularly if it was integrated within the clinical record, for: 1) always bearing in mind the need to consider the possibility of IPV, and 2) guidance on detection, inquiry and, to a certain extent, response. However, in order to be useful, the protocol has to be known, read and used by the professional—not just stored in the library, as happened in Rios. Moreover, a lack of follow-up and support might diminish the impact of the protocol on teams’ responses to IPV, as a nurse from Indias stated:

*When the protocol was launched and integrated in the clinical record*, *we all got into the protocol and followed it*, *but later on*, *this has faded due to all those issues that I told you about [lack of continuation of training activities*, *lack of follow up and support]*. *It has worn away; we lack resources and skills (Nurse 1*, *Indias*, *Cantabria)*

During the interviews, the professionals indicated that the protocol alone was not sufficient to deal with such a complicated issue. This was also highlighted by regional health system managers in Spain, who considered that responding to IPV required professionals to go beyond following the steps dictated in a protocol [16]. The solution formula also evidences that the team must use the protocol, but needs to combine it with other conditions in order to ensure good practices: experience in primary health care, training, the presence of a champion social worker or a good team climate. The literature also evidences that protocolised care can be useful in health services, but that its usefulness depends on a number of aspects dependent on both the intervention to be protocolised and the characteristics of the professionals and teams [46].

In terms of team factors not directly dependent on the intervention, the experience of working in primary health care was the most relevant. The relevance of the primary health care approach as a facilitator for the integration of an IPV response was also mentioned in the qualitative interviews. Having more experience in primary health care (in contrast to specialised care) better prepared professionals to deal with complex issues, to manage uncertainty, to build a trust relationship, and to take an holistic approach, and it made them more prone to work in teams.

*The advantage that we have in primary health care is continuity and trust*. *That’s something that I told people attending the course [she organised a training course on clinical interviews exploring IPV]*: *There is no hurry*, *unless it’s something very dangerous*. *I think that*, *first of all*, *you need to establish a trust relationship*, *and when you establish such a relationship*, *then there is hardly anything that you cannot ask (Family Physician 1*, *General*, *Cantabria)*.

Primary health care services are the entry point into the health system; they are more accessible than specialised services, and they offer continuity of care, an integral approach, coordination within the service, other health care services and other sectors (i.e. education, social services), a family approach and a community orientation [47–49]. Such an approach facilitates the integration of non-biomedical interventions, such as a health-care response to IPV, although evidence of the facilitating role of a primary health care approach to integrate a health-care response to IPV remains limited.

A good team climate, training and having a social worker with expertise on IPV appeared in three of the combinations. In the interviews, some clues were found of how a good team climate could lead to a better understanding of the victims and a more individualised approach that could trigger the best response; for instance, the existence of a favourable team climate allows a discussion of the cases to learn and better respond to women exposed to IPV.

However, the qualitative interviews and the observation also highlighted that team work is far from institutionalised within primary health care centres in Spain. Although such centres should work as multidisciplinary teams, team work has not been promoted, either for IPV or for other health issues: Consequently, the individual responses of health care professionals might be very heterogeneous within the same team:

*I think that the word team is problematic*. *[…] A team is not an entity; it’s integrated by professionals with very different sensitivities […] How each individual professional responds can be very different […] (Family Physician 2*, *General*, *Cantabria)*

The literature shows that training health care professionals about IPV through educational interventions that involve their own participation and experiential learning leads to increased perceived self-efficacy and knowledge, and that it triggers changes in their attitudes [50]. The relatively minor role of training in the solution formula does not align with the literature or with the perceptions of the professionals regarding the importance and quality of the training provided. The professionals considered that the training implemented in their regions, although it might have failed to reach the majority of professionals (since participation was voluntary in all but one of the regions explored), was perceived to be of good quality and transformative in terms of the way they perceived IPV:

*I think that the training course we had opened our eyes to this problem […] It allowed us to better understand women exposed to IPV…; I mean*, *it is not really possible to understand them as an outsider*, *but at least to better understand the cycle of violence they are going through (Family doctor 1*, *Salinas*, *Cantabria)*

Three aspects pointed out in the interviews could explain this contradiction. First, when this study was conducted, training had been discontinued or downgraded for a number of years. This might have had a yo-yo effect, increasing frustration among professionals:

*The course was good*, *but these issues [IPV] are very problematic; we are opening Pandora’s box*, *and I think that more training is needed […] After we received this training*, *we were very motivated*, *but later on*, *it has faded*, *because it was never repeated*, *never ever (PHCC Coordinator*, *Indias*, *Cantabria)*

Second, training might have contributed to enhanced knowledge and sensitisation, but it might not have had such a great impact on actual response, due to its complexity and the fact that the focus of the training might have been more on detection than on responses. Third, informal in-service training conducted by team members might not have been considered by the professionals to be “formal training” (as measured in the questionnaire), although it might have been just as relevant (or more) for shaping practices. This quotation from a family doctor in La Virgen exemplifies both points:

*The courses focus on detection; this is the focus in PHCC*. *What do we have to do*? *We have to detect cases […] But once you have detected them*, *what then*? *[…] This is not taught in the normal courses*. *However*, *our internal sessions [organised monthly in the health centre to discuss IPV cases] have helped us with this*, *by making us learn how to accompany the woman through this process (Family Physician 3*, *La Virgen*, *Murcia)*

The relevance of having a social worker that champions the IPV response was noted in the interviews. Social workers in PHCCs deal with “social issues” and are key in establishing referral linkages with other institutions [51]. As the interviewed professionals stated, social workers, especially when they were interested and knowledgeable about IPV, were influential and inspiring:

*Yes*, *she is the one who motivates us [the social worker]*.*[…] She is the one who informs us about developments in terms of programmes*, *courses […] Each year*, *she organises at least one session about the topic in the centre*, *to remind us*, *to inform us about new programmes (Family Physician 1*, *Castillo*, *C Valenciana)*

The qualitative information also aligned with the fzQCA that the presence of a social worker with knowledge and motivation about IPV alone does no suffice to trigger a good team response to IPV. Other factors matter, as the social worker from El Campo described in relation to her failure to inspire another PHCC team (Zarzas) where she worked:

*[The type of team response to IPV] depends on many issues*. *It’s […] not just about me making a change here [in El Campo]*. *I sometimes wonder why [the differences in responses to IPV between Zarzas and El Campo exist]*, *since I am the same person in both PHCCs*. *I think it has to do with the team; the team in El Campo is experienced in the biopsychosocial approach*, *in working as a multidisciplinary team*, *together with the social worker*. *The team in Zarzas is much less experienced…; in Zarzas*, *there are a number of doctors who came from the hospital to “retire” in the PHCC*, *with a strong biomedical training…[…] With this type of environment*, *there is a certain disparagement towards those issues…; they do not consider them as being related to health (Social worker*, *El Campo*, *Murcia)*

Finally, it is important to note that the fact that the solution formula did not cover all of the cases with good responses to IPV highlights that there are other conditions that are key to explaining how a good team response to IPV is generated within primary health care teams which were not explored in this study. The relevance of individual motivation, grounded on personal experiences and ideology, emerged strongly in the interviews, and it could be interesting to explore its impact in further studies.

*I think it depends on each individual*. *[…] I think that [with IPV]*, *it depends on whether or not you believe [that it´s a problem]*, *nothing else… I mean*, *if we are told that from now onwards*, *heparine control will have to be done in the PHCC*, *people might protest*. *They will say that they are not prepared*, *but at the end*, *they will do it*. *But with IPV*, *it doesn´t work like that… […] It has to do with sensitivity*, *psychology*, *personal interest… it has to do with deeply rooted attitudes and values… A professional that has been raised in a machista environment and has not challenged that structure that still believes that it´s his wife who has to do the laundry*, *keep the house clean…*, *a protocol is not going to change such a professional… (Midwife*, *Avecilla*, *Castilla-León)*

### Strengths and Limitations

We consider that the information presented in this study will be useful to improve interventions aimed at implementing a health care response to IPV, because changes generated at the team level: 1) are more sustainable than changes generated at the individual level, and 2) they contribute more to consistency, because the response that women will receive will be less dependent on the individual professional she meets. However, there are also limitations that should be pointed out. In terms of the conditions assessed, not all of the contextual factors and mechanisms contained in the programme theory were included in the qualitative comparative analysis due to: 1) none of the cases exhibited the condition (i.e. monitoring), 2) the potential relevance of such condition emerged during the process of data collection (i.e. intrinsic motivation), 3) difficulties in measuring them—i.e. more direct measurements of the application of a primary health-care approach.

In addition, the team conditions were mainly calculated as aggregated individual responses. Finally, although the focus of the study was on team level factors, during the data collection, it became clear (and we also noted it in the Results and Discussion section) that team work, within primary health care teams in Spain, is not implemented as well in reality as it is proclaimed in the policies and guidelines, with individual level and mini-team level factors probably playing a much stronger role.

## Conclusion

The analysis emerging from the 15 primary health care teams studied allowed us to revise the programme theory and reformulated it in the following terms:

Primary health care teams that perceived themselves as well prepared to deal with IPV, consider themselves as self-efficient to deal with IPV and have a woman-centred approach to IPV (measured in terms of their opinions in regards to their understanding of the victims) respond better to women exposed to IPV. These three mechanisms are necessary in order to trigger a better response, but they are not the only ones- there might be others that would trigger a good IPV response.

In terms of the capability of contextual factors—at the team level, in general, and in regards to the intervention- the use of the protocol and accumulated experience in PHC seem to be the most relevant contextual factors in triggering a good response, but in order to do so, they must be combined with other team factors—team climate, having a champion SW and training.

## Supporting Information

S1 Table Cases studied. Characteristics of the 15 cases explored.
(PDF)

S2 Table PREMIS questionnaires. Excel file with data from PREMIS questionnaires.
(XLS)

S3 Table Truth table. (PDF)
