# Correction: Mechanisms that Trigger a Good Health-Care Response to Intimate Partner Violence in Spain. Combining Realist Evaluation and Qualitative Comparative Analysis Approaches

**DOI:** 10.1371/journal.pone.0139184

**Published:** 2015-09-23

**Authors:** Isabel Goicolea, Carmen Vives-Cases, Anna-Karin Hurtig, Bruno Marchal, Erica Briones-Vozmediano, Laura Otero-García, Marta García-Quinto, Miguel San Sebastian

The affiliation is missing for the sixth author. Laura Otero-García is affiliated with #4 and #6.

There are errors in affiliation 4 for authors Carmen Vives-Cases and Laura Otero-García and in affiliation 6 for author Laura Otero-García. Affiliation 4 should be: CIBER de Epidemiología y Salud Pública (CIBERESP), Madrid, Spain. Affiliation 6 should be: Nursing Section, Faculty of Medicine, Universidad Autónoma de Madrid, Madrid, Spain.
