# Supplementary material for: Mechanisms that Trigger a Good Health-Care Response to Intimate Partner Violence in Spain. Combining Realist Evaluation and Qualitative Comparative Analysis Approaches
Source: PLoS One. 2015 Aug 13;10(8):e0135167. doi: 10.1371/journal.pone.0135167 (PMC4536036; doi:10.1371/journal.pone.0135167)
Supplement: S1 Table — Characteristics of the 15 cases explored. (PDF) [file pone.0135167.s001.pdf]

S1 Table. Cases studied. Characteristics of the 15 cases explored.

REGION	HEALTH CENTRE	No health professionals	Location	Neighbourhood	Other characteristics
CANTABRIA	General	38	Urban-capital	Mixture of areas of high socioeconomic status and others of very low status.	Some medical doctors acknowledged as national experts on "patient doctor" communication.<br>Some professionals have been on the team for many years, while others are new- an important number coming from hospital practice.<br>Good relationship within the team and with the community.<br>Providers interested in doing a good job in their consultations, but not in other extra activities. No preventive activities.<br>They do not have a medical coordinator, as a conscious decision, but they consider that they manage well.
	Vegas	24	Urban- no capital	An industrial area strongly hit by unemployment.	The team gets along well together; they have a room where they gather for coffee breaks and lunch, and there are social activities that they do together. Half of the staff is old, with 15-20 years in the health centre, while half is young.
	Salinas	16	Rural	Small town of middle low socioeconomic status.	Very good relationship with the community.<br>Three nurses visit the two high schools in the town twice a week and have consultations there on those two days. This has been running for several years now. Currently, one nurse has also started a therapeutic group for women.<br>The medical coordinator works in another health post, and team meetings are not regular.<br>Active team- Facebook page, weekly demonstrations against decreased funding for the public health system.
	Indias	13	Rural	Small rural village.	Professionals working on the team also live in the village or neighbouring villages, and they know their patients as neighbours or friends.<br>Small team that gets along very well together.<br>There are satellite health posts, but all of them meet every Thursday (with the exception of the social worker, who works at another PHC on that day).

CASTILLA-LEON	Mares	22	Urban-regional capital	Located in the city centre, in a middle-high socioeconomic neighbourhood.	In general, there is a good relationship between patients and health care providers.<br>The person who is now in charge of the IPV program in the RHS worked there some years ago.<br>The centre focuses on curative services, patient-centred approach only among selected professionals, and there are no preventive activities.
	Angeles	26	Urban regional capital	Located in the outskirts of the city in a middle low socioeconomic neighbourhood.	Not a strong relationship between the HC and the community.<br>Focus on curative services.<br>There is no medical coordinator at the moment. They mention that beforehand they used to work more as a team, but currently, this is no longer possible due to work pressure (fewer substitutions and fewer staff due to austerity measures).
	Avecilla	23	Rural	Rural small village with aging population. Population density is low, and people are distributed across a number of small villages with small populations.	The health professionals have to visit several small villages, and the health centre in AG works as a meeting centre.<br>They attend mainly old and very old people, as well as some tourists during the summer.<br>Medical coordinator has a good relationship with the health professionals.<br>Midwife is a regional trainer on IPV and runs therapeutic groups for women.

MURCIA	La Virgen	35	Urban capital	Middle-low socioeconomic neighbourhood.	Good relationship with the community; they run a number of community groups where they engage in prevention and health promotion activities. There is team work in this health centre; especially in terms of IPV, there is team work among a number of GPs, nurses, the resident psychologist, the social worker and some paediatricians. Midwives are not involved. Health centre with a strong PHC approach (a role model for this in the region and beyond). Also actively involved in training programme on the women's malaise approach.<br>Several team members acknowledged regionally as experts on IPV response.
	El Campo	21	Rural	Health centre in a rural agricultural area. Small town where people know each other, which makes it more difficult to disclose cases and file denounces.	Good relationship with patients. They know each other.<br>Social worker very motivated and an expert on IPV (previously in charge of that programme within the RHS), and also a midwife and one GP.<br>Biopsychosocial approach used by many GPs, less among nurses. Social worker and midwife implement a women's malaise group, and they have been involved in developing different plans and protocols related to IPV at the regional level.
	Mora	33	Rural	1 slightly larger HC and satellite health posts.	People go to the HC due to biological problems, and it is difficult for them to consider going to the HC to disclose IPV.<br>There is not a team style of responding to IPV; each professional does the best that he/she can. They do not share information about those cases. The majority consider responding to IPV as responding to obvious cases, but not as the early detection of cases.<br>Since they have good relationships with patients, issues of IPV might be mentioned directly by women, especially physical IPV, and they will issue legal reports, recommend denouncing and/or refer to the social worker.

	Cristina	32	Urban capital	High-middle class neighbourhood.	No team work in this health centre.<br>One of the persons who is an expert on IPV and who is currently working at the managerial level in Murcia was a GP in this centre. Many acknowledge her as a key person regarding this topic, but her expertise has not been inherited by any of the professionals currently working in Cristina. This is also a health centre where women and children who are in shelters come to be attended.
C VALENCIANA	Santos	33	Urban- provincial capital	Middle-level socioeconomic neighbourhood	Some community work with schools on sex education done by two nurses.<br>One family physician is the coordinator for teaching and learning; he is very active in promoting meetings and workshops in the health centre.
	Rios	29	Small town in rural agricultural area	Middle-low socioeconomic status.	Health centre located in a health area under private management.<br>They do not have a social worker.<br>Very active department of public health.<br>Community project on IPV running, and previously, there was another project with institutions partly funded by an EU grant.
	Castillo	25	Small town in rural artisans/agricultural area	Middle-low socioeconomic status. Very active town in terms of social participation.	Social worker and sexologist are very active in working on issues related to violence against women. They have a women´s group and a group with women who have been victims of sexual abuse There is a reference network between the health centre, the police, the municipal services and the judicial system to support women who have been exposed to IPV.
	Naranjo	28	Urban regional capital		Medical coordinator is an expert on IPV. She teaches other health professionals about this topic in the autonomous region and also teaches medical students at the University.<br>At this health centre, there is also a very active group pf physicians who do research on cardiovascular diseases, including clinical trials.
