# Supplementary material for: Mechanisms that Trigger a Good Health-Care Response to Intimate Partner Violence in Spain. Combining Realist Evaluation and Qualitative Comparative Analysis Approaches
Source: PLoS One. 2015 Aug 13;10(8):e0135167. doi: 10.1371/journal.pone.0135167 (PMC4536036; doi:10.1371/journal.pone.0135167)
Supplement: S3 Table — (PDF) [file pone.0135167.s003.pdf]

S3 Table. Truth table

Train	Prot	PHC	Clim	SW	SE	VU	Know	PPrep	TeamIPV	Pract	raw consist	pri consist
0	1	1	1	1	1	1	1	1	1	1	0.986	0.946
0	1	1	1	1	1	0	1	1	1	1	0.982	0.912
1	1	1	1	0	1	1	0	1	0	1	0.979	0.941
0	1	1	0	0	1	1	1	1	0	1	0.95	0.821
1	0	1	0	0	1	1	1	1	1	1	0.948	0.867
0	0	0	1	1	1	1	0	1	1	1	0.891	0.611
1	1	0	0	1	1	1	0	1	1	1	0.866	0.5
1	1	0	0	0	0	0	0	1	0	0	0.772	0
0	0	0	1	0	0	1	1	0	1	0	0.708	0
0	0	1	0	0	0	1	0	0	0	0	0.654	0
0	0	1	0	0	0	0	0	0	0	0	0.563	0
0	0	0	0	0	0	0	0	1	0	0	0.510	0
0	0	0	1	0	0	0	0	0	0	0	0.449	0
0	0	0	0	0	0	0	0	0	0	0	0.422	0
